# E-type prostanoid receptor 4 (EP4) in disease and therapy

**DOI:** 10.1016/j.pharmthera.2013.03.006

**Published:** 2013-06

**Authors:** Viktoria Konya, Gunther Marsche, Rufina Schuligoi, Akos Heinemann

**Affiliations:** Institute of Experimental and Clinical Pharmacology, Medical University of Graz, Austria

**Keywords:** AMPK, AMP-activated protein kinase, cAMP, cyclic adenylyl monophosphate, CFTR, cystic fibrosis transmembrane conductance regulator, ClC, chloride channel, COX, cyclooxygenase, CREB, cAMP-response element-binding protein, DP, D-type prostanoid receptor, DSS, dextran sodium sulfate, EGFR, epidermal growth factor receptor, eNOS, endothelial nitric oxide synthase, EP, E-type prostanoid receptor, Epac, exchange protein activated by cAMP, EPRAP, EP4 receptor-associated protein, ERK, extracellular signal-regulated kinase, FEM1a, feminization 1 homolog a, FP, F-type prostanoid receptor, GRK, G protein-coupled receptor kinase, 5-HETE, 5-hydroxyeicosatetraenoic acid, ICER, inducible cAMP early repressor, ICAM-1, intercellular adhesion molecule-1, Ig, immunoglobulin, IL, interleukin, IFN, interferon, IP, I-type prostanoid receptor, LPS, lipopolysaccharide, MAP, mitogen-activated protein kinase, MCP, monocyte chemoattractant protein, MEK, MAP kinase kinase, NF-κB, nuclear factor kappa-light-chain-enhancer of activated B cells, NSAID, non-steroidal anti-inflammatory drug, PG, prostaglandin, PI3K, phosphatidyl insositol 3-kinase, PK, protein kinase, TP, T-type prostanoid receptor, TX, thromboxane receptor, Prostaglandins, Inflammation, Vascular disease, Cancerogenesis, Renal function, Osteoporosis

## Abstract

The large variety of biological functions governed by prostaglandin (PG) E_2_ is mediated by signaling through four distinct E-type prostanoid (EP) receptors. The availability of mouse strains with genetic ablation of each EP receptor subtype and the development of selective EP agonists and antagonists have tremendously advanced our understanding of PGE_2_ as a physiologically and clinically relevant mediator. Moreover, studies using disease models revealed numerous conditions in which distinct EP receptors might be exploited therapeutically. In this context, the EP4 receptor is currently emerging as most versatile and promising among PGE_2_ receptors. Anti-inflammatory, anti-thrombotic and vasoprotective effects have been proposed for the EP4 receptor, along with its recently described unfavorable tumor-promoting and pro-angiogenic roles. A possible explanation for the diverse biological functions of EP4 might be the multiple signaling pathways switched on upon EP4 activation. The present review attempts to summarize the EP4 receptor-triggered signaling modules and the possible therapeutic applications of EP4-selective agonists and antagonists.

## 1 Introduction

Prostaglandins (PGs) and thromboxane A_2_ (TXA_2_) are synthesized from arachidonic acid by cyclooxygenase (COX) and specific prostanoid synthases. Released in response to various physiological and pathological stimuli they play essential roles in maintaining body homeostasis. Prostaglandin E_2_ (PGE_2_) is involved in several biological processes such as pain, fever, regulation of vascular tone, renal function, mucosal integrity, inflammation, angiogenesis and tumor growth. PGE_2_ often causes complex and divergent effects which can be attributed to its activation of four so-called E-type prostanoid receptors (EP1 to EP4). Studies conducted with EP receptor knock-out mice and the recent availability of highly selective pharmacological agonists and antagonists have allowed us to identify distinct — although sometimes overlapping — roles for EP receptor subtypes in PGE_2_-regulated processes. In this review, we summarize the physiological roles of EP4, its molecular structure and intracellular signaling pathways, and how EP4 receptors might be targeted pharmacologically for the benefit of human disease, including vascular and renal diseases, inflammation, osteoporosis and cancer. Tables 1–3 provide an overview of currently described selective and non-selective agonists, and antagonists for EP4, respectively, and their affinity, dosing and biological effects.

## 2 Prostaglandin E_2_ and its four receptors

**Fig. 1:**
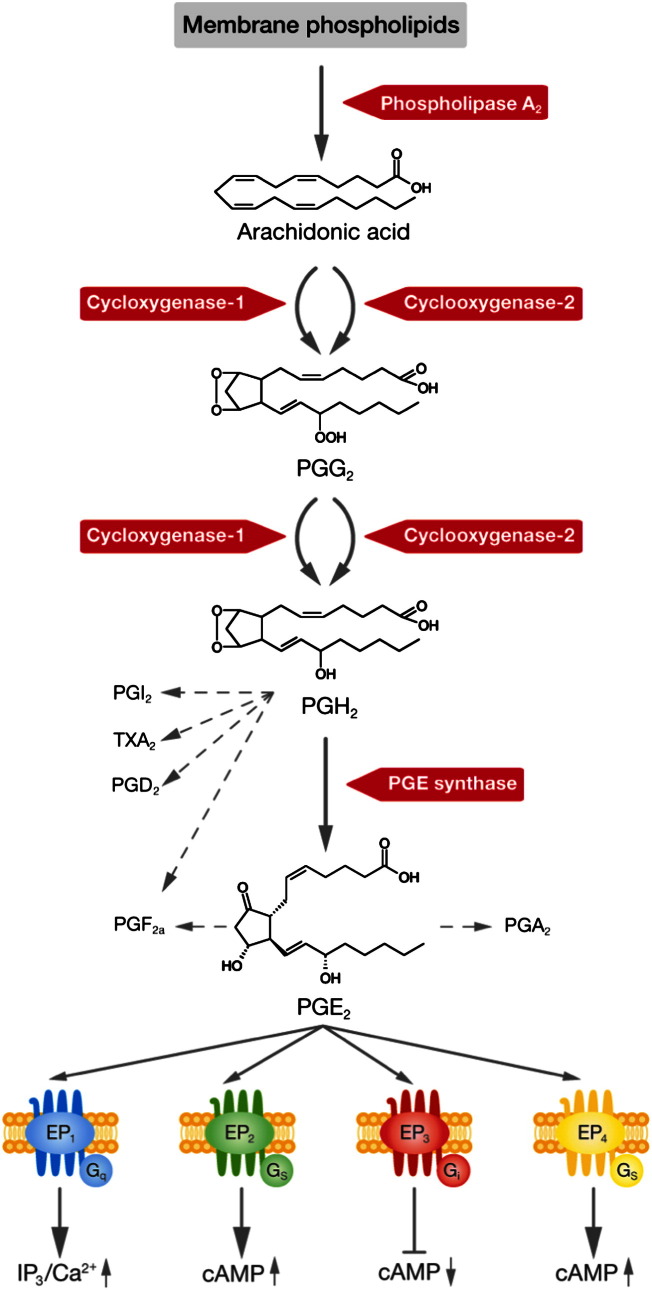
PGE_2_ biosynthesis and receptors. Arachidonic acid is liberated from membrane phospholipids by phospholipase A_2_ enzyme activity. Arachidonic acid is converted to the endoperoxide PGG_2_ and further reduced to PGH_2_ by the action of cyclooxygenase 1 and 2 enzymes. PGE_2_ is formed from PGH_2_ by PGE synthases and binds to and activates four EP receptor subtypes, designated EP1 to EP4 receptors. These receptors are coupled to different G proteins leading to subsequent activation of specific signal transduction pathways. Besides PGE_2_, other prostanoids are also formed from PGH_2_, i.e. PGI_2_, TXA_2_, PGD_2_ and PGF_2α_. Additionally, PGE_2_ can also be converted to PGA_2_.

Prostanoids are derived from arachidonic acid, a 20 carbon polyunsaturated fatty acid, which is usually found in phosphoglycerides of mammalian cell membranes (Fig. 1). Arachidonic acid is released by phospholipase A_2_ from the cell membrane and is converted to PGG_2_ and then reduced to PGH_2_ by COX-1 or COX-2 (Smith & Dewitt, 1996; Park & Christman, 2006). In general COX-1 is constitutively active whose expression appears to be regulated developmentally, while COX-2 is usually absent from cells but its expression can be induced in response to different stimuli. However, COX-2 is also constitutively expressed in some tissues, such as the endothelium, kidney, gastrointestinal mucosa and brain (O'Banion, 1999; Grosser et al., 2006; Zidar et al., 2009). The endoperoxide intermediate PGH_2_ is converted to the different prostanoids (PGE_2_, PGI_2_, TXA_2_, PGD_2_, PGF_2α_) by specific synthases. In general, the expression pattern of the prostanoid synthases is specific for different cells and determines which prostanoid they will be producing in abundance. PGE_2_ is generated at large amounts in fibroblasts, monocytes, and epithelial and endothelial cells by three enzyme isoforms, i.e. inducible microsomal PGE synthase-1, and constitutively expressed microsomal PGE synthase-2 and cytosolic PGE synthase (Fig. 1) (Kudo & Murakami, 2005).

The various biological effects of PGE_2_ are mediated by four EP receptors, which show differential patterns of tissue distribution. EP1 mRNA is ubiquitously expressed in murine tissues, while EP3 receptor mRNA levels are high in adipose tissues, pancreas, kidney and vena cava. EP4 mRNA is mainly expressed in the gastrointestinal tract, uterus, hematopietic tissues and skin, whereas EP2 receptor mRNA was found to be least abundant among EP receptors, with the highest expression occurring in the airways, ovary, bone marrow and olfactory epithelium (Regard et al., 2008). The EP receptors are coupled to different G proteins and accordingly activate diverse signaling pathways (Fig. 1) (Alfranca et al., 2006). EP1 signals predominantly via G_q_, thereby elevating intracellular Ca^2+^ levels through the action of phospholipase Cβ followed by activation of protein kinase (PK) C. EP2 and EP4 are described as G_s_ coupled receptors, which activate adenylate cyclase and induce intracellular cyclic adenylyl monophosphate (cAMP) production, while EP3 is coupled to G_i_ and inhibits cAMP production (Alfranca et al., 2006). The human EP1 receptor is comprised of 402 amino acids, the EP2 from 358, the EP3 from 390 while the EP4 receptor is composed of 488 amino acids, additionally there are two alternative splice variants of the EP1 and eight variants of the EP3 (Regan, 2003). A similar tendency can be observed in murine EP1, EP2, EP3 and EP4 receptors, which consist of 405, 362, 366 and 513 amino acids, respectively (Negishi et al., 1993; Sugimoto & Narumiya, 2007).

## 3 E-type prostanoid receptor 4 structure, signaling and ligands

### 3.1 Structure

When first cloned in 1993 as a PGE_2_ receptor that stimulated cAMP formation, the EP4 receptor was designated as “EP2” (Honda et al., 1993). After another cAMP-stimulating PGE_2_ receptor had been discovered which was sensitive to butaprost (Regan et al., 1994), the butaprost-insensitive receptor which mediated vasorelaxation in piglet saphenous vein was renamed “EP4” (Coleman et al., 1994; Nishigaki et al., 1995; Breyer et al., 1996).

**Fig. 2:**
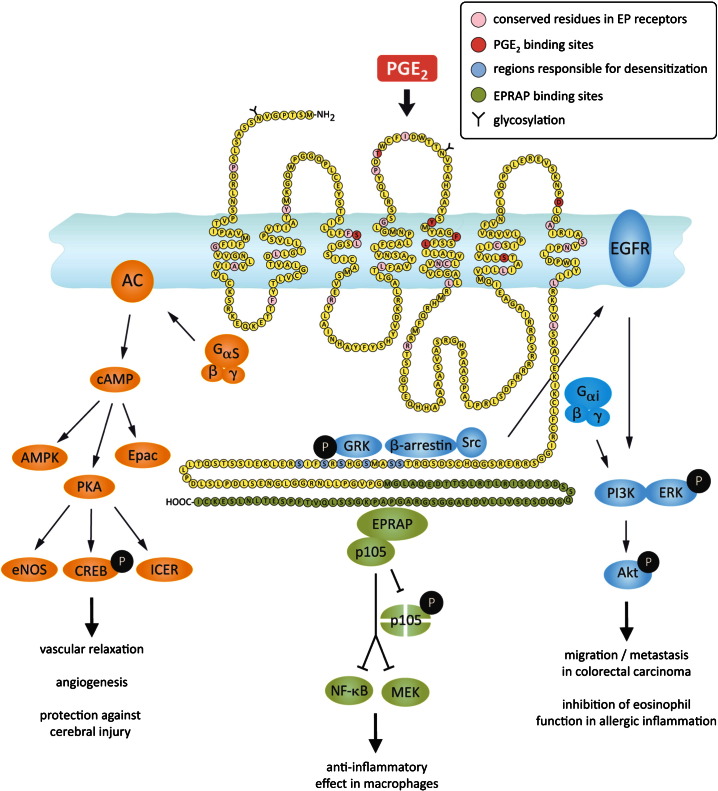
The structure of the EP4 receptor and its activation of signaling modules. The 488 amino acid sequence of the human EP4 receptor is displayed with color code indicating the single residues conserved in all EPs, the binding sites for the native ligand PGE_2_, regions responsible for desensitization and for interaction with EPRAP. Binding of an agonist induces G_αs_-dependent activation of adenylate cyclase (AC), formation of cAMP and either activation of Epac (exchange factor activated by cAMP), activation of the PKA-independent AMP-activated protein kinase (AMPK) or activation of PKA and eNOS, or alternatively the transcription factor CREB (cAMP-responsive element-binding protein). Additionally, cAMP can act through inducible cAMP early repressor (ICER). EP4 receptor activation has been found to reduce retinoic acid secretion by ICER-mediated blockade of the expression of retinoic acid dehydrogenase These signals finally mediate vascular relaxation, angiogenesis and protection against cerebral ischemic injury. EP4 receptor becomes rapidly desensitized upon binding of G protein-coupled receptor kinases (GRK) to serine residues on the C-terminus. These residues are phosphorylated and attract β-arrestin initiating receptor internalization, and c-Src which leads to transactivation of EGFR and further activation of PI3K/ERK/Akt kinases. Alternatively, Pertussis toxin-sensitive G_αi_ can be activated which also may induce the activation of PI3K/ERK pathway. These latter signaling pathways enable migration and metastasis of colorectal carcinoma and inhibit the activation of eosinophils in allergic inflammation. The extended C-terminus of EP4 receptor allows interaction with EPRAP which in turn stabilizes the p105 subunit that prevents the activation of NF-κB and mitogen-activated protein kinase kinase/extracellular signal-regulated kinase kinase 1/2 (MEK/ERK1/2), and inhibits transcription of pro-inflammatory cytokines in activated macrophages.

The EP4 gene contains three exons of which the first is non-coding. The initiation site was found to lack a TATA box, but it is GC-rich, and contains two CCAAT boxes and SP1-, AP2- and NF-κB-binding motifs (Foord et al., 1996). Across EP receptors in humans and mice, most amino acid conservation can be found in the transmembrane helices which form the binding pocket for the natural ligand PGE_2_ (Fig. 2) (Stillman et al., 1998). Amino acid homology among the EP receptor subtypes is small, between single EP receptors not more than 30%. Even EP2 and EP4 receptors, which are both coupled to G_αs_ proteins and activate adenylate cyclase, share only 38% homology in humans and 31% in mice (Regan, 2003; Sugimoto & Narumiya, 2007). Regarding the interaction of PGE_2_ with the EP4 receptor, a single threonine residue, T168, in the second extracellular loop was identified as being essential (Fig. 2) (Stillman et al., 1998). Furthermore, the very recent 3D modeling of the human EP4 receptor and in silico docking experiments for PGE_2_ revealed the most likely binding sites of PGE_2_ on the 3D structure of EP4. These pertinent amino acid residues are S103, T168, Y186, F191, L195, S285, and D311 (Fig. 2) (Margan et al., 2012). Radioligand binding studies suggested that the affinity of PGE_2_ for EP4 receptors is higher (EC_50_: 2.8 nM) than that for EP2 (EC_50_: 19 nM) (Fujino et al., 2002).

### 3.2 Signaling pathways

The EP4 receptor was initially described as a G_αs_ protein-coupled receptor leading to stimulation of adenylate cyclase and elevation of intracellular cAMP levels (Coleman et al., 1994). EP4 stimulation, among other functions, induces vascular relaxation mediated by cAMP, PKA and endothelial nitric oxide synthase (eNOS) (Hristovska et al., 2007), and angiogenesis via cAMP and PKA Cμ (Zhang & Daaka, 2011) (Fig. 2). The transcription factor cAMP-responsive element-binding protein (CREB) can be phosphorylated by PKA or different MAP kinases (Johannessen et al., 2004; Johannessen & Moens, 2007). CREB regulates the expression of FcγRIIA, an important receptor of neutrophils and monocytes in combating bacterial infections. Endogenous or exogenously added PGE_2_ activates CREB through PKA in the differentiation of the myeloid PLB cell line, an effect that depends on EP4 receptors (Hazan-Eitan et al., 2006). In monocytes, EP4 receptor-mediated CREB activation and binding to the promotor of the chemokine receptor CCR7 enhances the expression of the receptor and augments the migration of the cells (Cote et al., 2009). Alternatively, cAMP can act through inducible cAMP early repressor (ICER), a truncated CREB analog that binds to cAMP response-element but blocks transcription, as it lacks the trans-activation domain. EP4 receptor activation has been found to reduce retinoic acid secretion by ICER-mediated blockade of the expression of retinoic acid dehydrogenase (Stock et al., 2011).

An additional EP4 receptor signaling molecule independent of PKA is Epac, i.e. the exchange protein activated by cAMP. Epac-1 and Epac-2 are guanine nucleotide-exchange factors that link cAMP and members of the Ras superfamily such as Rho, Rac and Ras. As such, Epac signaling is generally involved in cell proliferation, differentiation, migration, and inflammatory responses (Bos, 2003). Epac and PKA can signal synergistically, as shown for the closure of ductus arteriosus (Yokoyama et al., 2008). Finally, EP4 receptors in podocytes signal to induce COX-2 through an indirectly cAMP-dependent, PKA-independent signaling pathway: AMP-activated protein kinase (AMPK) (Faour et al., 2008). Therefore, PGE_2_–EP4 signaling can activate multiple cAMP-dependent pathways, including PKA, Epac and AMPK, which might act in concert or in alternating manner to mediate G_αs_-dependent effects of the EP4 receptor.

Moreover, its C-terminus exhibits potential phosphorylation sites for PKA and G protein-coupled receptor kinases (GRK), and might provide additional interaction sites for other signaling molecules such as arrestins or EP4 receptor-associated protein (EPRAP) (Desai & Ashby, 2001; Regan, 2003; Takayama et al., 2006). Most probably, these signaling pathways are activated in parallel and explain the much weaker cAMP response to PGE_2_ in EP4 receptor-transfected cells as compared to EP2-transfected cells (Fujino et al., 2002). Additionally, EP4 receptor-overexpressing CHO cells showed rapid desensitization and internalization of EP4 after PGE_2_ stimulation, which was not observed with EP2 receptors (Nishigaki et al., 1996). Furthermore, differential N-glycosylation also influences the maintenance of receptor surface expression (Ludwig et al., 2000). EP4 receptor has two putative N-glycosylation sites, while EP2 has four. The amino acid residues in the EP4 receptor responsible for desensitization were identified on the long C-terminal tail as six serine residues in positions 370, 371, 374, 377, 379, 382. These residues are potential targets of phosphorylation by PKA and GRK (Fig. 2) (Bastepe & Ashby, 1999; Desai et al., 2000). The phosphorylation of EP4 receptor recruits β-arrestin-1 which in turn activates c-Src to initiate the transactivation of the epidermal growth factor receptor (EGFR) and subsequent downstream signaling through phosphatidyl insositol 3-kinase (PI3K) and Akt (Buchanan et al., 2006). The activation of this signaling cascade has been proposed to regulate the migration and metastasis of colorectal carcinomas (Fig. 2) (Buchanan et al., 2006).

EP4 receptor can additionally couple to the Pertussis toxin-sensitive inhibitory G-protein (G_αi_) that also leads to activation of PI3K/ERK signaling in EP4 receptors overexpressed in HEK293 cells (Fig. 2) (Fujino et al., 2003; Fujino & Regan, 2006). EP4 receptor ligation induced in vivo neurovascularization in mice, and in vitro endothelial migration and tube formation via ERK signaling (Rao et al., 2007). EP4 receptor-mediated protection in a cerebral ischemia mouse model was found to depend on the Akt/eNOS pathway (Liang et al., 2011). In addition, a recent report from our lab demonstrated that inhibition of eosinophil function by an EP4 receptor-selective agonist (ONO AE1-329) depends on PI3K/PKC but not on cAMP/PKA (Luschnig-Schratl et al., 2011).

Further increasing the complexity of EP4 receptor signaling, a binding site for EPRAP has been identified on the long cytoplasmic tail of EP4 in human macrophages (Takayama et al., 2006). EPRAP was shown to stabilize the p105 subunit of NF-κB that in turn prevents the activation of NF-κB and mitogen-activated protein kinase kinase/extracellular signal-regulated kinase kinase 1/2 (MEK/ERK1/2), and further inhibits the transcription of pro-inflammatory cytokines (Fig. 2) (Minami et al., 2008). Utilizing this G protein-independent mechanism, EP4 receptors play an anti-inflammatory role in human and murine macrophages and might inhibit the proliferation of a mouse immature B lymphocyte cell line (Takayama et al., 2006; Minami et al., 2008; Prijatelj et al., 2012).

### 3.3 Ligand-based signaling pathways

In view of the distinct signaling pathways originating from EP4, the question arises whether different EP4 receptor ligands may differentially trigger these signaling modules. The first study to systematically compare the activation of G_αs_, G_αi_ and β-arrestin signaling pathways revealed characteristic differences between various ligands and native PGE_2_ (Leduc et al., 2009). In bioluminescence resonance energy transfer (BRET) assays investigating HEK293 cells overexpressing EP4 receptor, PGE_2_ activated G_αs_ with 10-fold higher potency over G_αi_, while other ligands, such as the EP4 receptor agonists L-902688 and PGE_1_-OH, were biased towards G_αi_ and β-arrestin over G_αs_. All these responses were shown to be mediated by EP4 receptors using the EP4-specific antagonist GW627368X (Leduc et al., 2009). EP4 receptor-selective agonists and EP2/EP4 receptor agonists are listed in Tables 1 and 2, respectively. EP4 receptor-selective antagonists are shown in Table 3.

## 4 Immune modulation by E-type prostanoid receptor 4

COX inhibitors, also referred to as non-steroidal anti-inflammatory drugs (NSAIDs), are widely used for symptomatic treatment of inflammatory diseases, particularly due to their potent analgesic effect. However, their clinical use is considerably limited as they give rise to gastrointestinal, renal and cardiovascular complications, among others. Selective COX-2 inhibitors that had been developed to minimize adverse effects by sparing the homeostatic functions of COX-1 did not meet expectations due to increased cardiovascular risk (Grosser et al., 2010). This Janus head nature of prostaglandins is consistent with the numerous pro- and anti-inflammatory effects that PGE_2_ can exert, which might be up to local concentrations of PGE_2_ and expression patterns of its receptors. The anti-inflammatory actions of PGE_2_ have been mainly ascribed to the EP4 receptor (Tang et al., 2012), although the EP2 receptor may play similar, redundant roles in many cellular and in vivo models. Nevertheless, the EP4 receptor might also act to promote inflammation, particularly in Th17-dependent diseases (Sheibanie et al., 2007; Sakata et al., 2010), and is widely considered as an important pro-tumorigenic molecule (Chell et al., 2006).

The EP4 receptor is expressed on most immune cell types, and functional responses mediated by EP4 receptors have been reported in B- and T-lymphocytes, natural killer cells, dendritic cells, eosinophils, monocytes and macrophages (Norel, 2007; Kalinski, 2012). PGE_2_ and EP4 receptors are indispensable for monocyte-derived dendritic cells and Langerhans cells to attain a migratory phenotype (Luft et al., 2002; Kabashima et al., 2003), which is a prerequisite for them to evade into lymph nodes and subsequently present antigens there. EP2/EP4-mediated expression of matrix metalloprotease-9 in dendritic cells appears to be crucial for their migration (Yen et al., 2011). Concomitantly, cytokine release from these cells is inhibited while survival is enhanced by the EP4 receptor (Luft et al., 2002; Jing et al., 2003; Baratelli et al., 2005).

Recently, PGE_2_ was recognized to positively modulate the differentiation and activity of Th17 cells. EP4 receptor stimulation accentuates LPS-induced IL-23 secretion from immature dendritic cells and supernatants from these cells enhance IL-17 production in activated T cells (Sheibanie et al., 2007). Naïve T cells upregulate IL-23 and IL-1 receptors in response to EP2/EP4 signaling and PGE_2_ synergizes with these cytokines in promoting the typical Th2 phenotype, whereby EP4 receptor stimulation particularly leads to IL-10 downregulation (Boniface et al., 2009; Napolitani et al., 2009). In vivo, the EP4 antagonist ONO-AE3-208 ameliorated experimental autoimmune encephalomyelitis or contact hypersensitivity, and reduced IL-17 production in regional lymph nodes (Yao et al., 2009). In contrast, human plasmacytoid dendritic cells produce less interferon-α following EP2/EP4 stimulation by PGE_2_ and lose their Th1 co-stimulatory capacity (Fabricius et al., 2010).

Dendritic cells that mature in the presence of PGE_2_ preferably drive Th2 polarization, and produce Th2-attracting chemokines, which might argue for a crucial role of EP4 receptors in allergic sensitization (Kubo et al., 2004; McIlroy et al., 2006). This notion is further supported by the observation that PGE_2_ promotes the differentiation of B-cells and the class-switch to IgE through EP2 and EP4 receptors (Fedyk & Phipps, 1996). In contrast, PGE_2_ suppresses antigen-specific responses, such as proliferation and cytokine secretion of human Th1- and Th2 cells through EP2/EP4 signals (Okano et al., 2006), suggesting that PGE_2_ might limit allergic responses upon allergen re-exposure. PGE_2_ might also take part in systemic immune suppression following UV irradiation of the skin, an effect that was due to accumulation of self-tolerant, DEC205-expressing, dendritic cells and CD4^+^/Foxp3^+^ regulatory T cells in peripheral lymph nodes, but was not observed in mice treated with an EP4 antagonist (Soontrapa et al., 2011).

### 4.1 Macrophages, atherosclerosis and sepsis

PGE_2_ has a dual effect on macrophages; on the one hand stimulating their motility (Tajima et al., 2008), on the other hand suppressing the release of inflammatory chemokines. Macrophages stimulated with LPS and pro-inflammatory cytokines, such as TNF-α, IFN-γ and IL1-β showed markedly reduced secretion of IL-8, MCP-1, MIP-1α and β and IP-10 upon PGE_2_ pretreatment (Takayama et al., 2002). Macrophages express the EP4 receptor, and all these anti-inflammatory effects of PGE_2_ were suppressed by the selective EP4 antagonist L-161982. However, this anti-inflammatory response to PGE_2_ was not mediated by the cAMP/PKA/CREB pathway. The signaling pathway involved was identified to include a novel cytoplasmic signaling partner of the EP4 receptor, designated as EP4 receptor-associated protein (EPRAP) (Takayama et al., 2006). In human atherosclerotic plaques, EPRAP co-localized with the EP4 receptor, and siRNA targeting of EPRAP limited the PGE_2_-induced anti-inflammatory effects in macrophages, while the PGE_2_-induced cAMP increase was not significantly affected. EPRAP shows the highest degree of homology with the murine feminization 1 homolog a (Fem1a), which is a homolog of the *Caenorhabditis elegans* FEM-1 protein. The FEM-1 protein is involved in the regulation of transcription factors in the sex-determination cascade of *C. elegans*. Furthermore, EPRAP contains 8 sequential ankyrin repeats, which suggests its potential involvement in protein–protein interaction. Such ankyrin repeats can be found e.g. in nuclear factor (NF)-κB and IκB (Malek et al., 1998). The actual interaction of EP4 receptor and EPRAP with NF-κB was shown subsequently by the same group (Minami et al., 2008). In macrophages, the LPS-induced NF-κB activation was observed to be blocked by PGE_2_. In detail, LPS treatment of macrophages induces the phosphorylation of NF-κB1 p105, which leads to its degradation, and in turn allows the activation of NF-κB and the subsequent transcription of pro-inflammatory genes. At this point, the EP4 receptor-associated EPRAP stabilizes the p105 subunit by preventing its phosphorylation and degradation, thereby inhibiting NF-κB and mitogen-activated protein kinase kinase 1/2 (MEK) in macrophages (Minami et al., 2008). EP4 receptor activation was also found to attenuate cytokine release from human alveolar macrophages (Ratcliffe et al., 2007). In a very similar manner, PGE_2_ acting via EP4 receptors attenuated the activation of microglia and prevented lipid peroxidation and proinflammatory gene expression in a murine model of LPS-induced brain inflammation (Shi et al., 2010).

Macrophages play an important role in lipid homeostasis in the vasculature, pertinent to atherosclerosis. The role of EP4 receptors was addressed by allogenic hematopoietic cell transplantation from mice deficient in EP4 receptors to animals lacking the low density lipoprotein receptor. EP4 deficiency in hematopoietic cells partially protected against early atherosclerotic lesions (Babaev et al., 2008), but enhanced the inflammation in advanced atherosclerotic plaques and facilitated the formation of angiotensin II-induced abdominal aortic aneurysms (Tang et al., 2011a, 2011b). In sharp contrast, systemic treatment of mice with the EP4 antagonist, ONO-AE3-208, or a heterozygous EP4^+/−^ genotype decreased vascular inflammation and protected from angiotensin II-induced abdominal aortic aneurysm formation on an ApoE-deficient background (Cao et al., 2012; Yokoyama et al., 2012). These observations might suggest that EP4 receptors in hematopoietic and somatic cells play opposing roles in vascular homeostasis.

Sepsis is characterized by uncontrolled activation of inflammatory cascades, often followed by a shift toward an immunosuppressive state (Hotchkiss & Karl, 2003). In a recent study, arachidonic acid metabolites like TXB_2_, 5-HETE and PGE_2_ were quantified using a sensitive mass spectrometry approach in whole blood samples of patients with severe sepsis (Bruegel et al., 2012). Most strikingly, PGE_2_ and PGE synthase levels were reduced in blood samples of septic patients, both at baseline and also following ex vivo stimulation with LPS. The positive regulatory role of PGE_2_ in sepsis was further supported by an increase of PGE_2_ release in patients with a favorable clinical course of the disease (Bruegel et al., 2012). However, the EP receptor mediating the protective role of PGE_2_ in sepsis has not yet been identified, but it is likely that EP4 receptor-mediated suppression of monocyte cytokine release plays a major role (Iwasaki et al., 2003).

A previous study, using a mouse sepsis model induced by cecal ligation and puncture, demonstrated that administration of bone marrow stromal cells suppressed macrophage activation by increasing the secretion of IL-10 and leading to amelioration of multi-organ inflammation. PGE_2_ was revealed to mediate this response via EP4 and EP2 receptors on macrophages (Nemeth et al., 2009). As such, the EP4 receptor and EPRAP might provide novel therapeutic targets in chronic inflammatory diseases with excess of macrophage activation, such as atherosclerosis and sepsis.

### 4.2 Eosinophils and allergic inflammation

Infiltration of eosinophils, a major effector cell type involved in allergic inflammation and asthma, was found to be markedly enhanced in COX-1 and COX-2 knockout mice (Gavett et al., 1999). This suggested a possible inhibitory effect of prostaglandins on eosinophils. In fact, stimulation of EP4 receptor by ONO AE1-329 and PGE_2_ effectively inhibited eosinophil function including chemotactic responses, CD11b adhesion molecule expression and formation of reactive oxygen species (Luschnig-Schratl et al., 2011). On the subcellular level, EP4 receptor activation resulted in blockade of cytoskeletal reorganization and inhibition of intracellular Ca^2+^ release. All these effects were reversed by antagonizing the EP4 receptor using GW627368x and ONO AE3-208. The EP4-mediated inhibition of eosinophil function depended on PI3K and PKC but not the cAMP/PKA pathway (Luschnig-Schratl et al., 2011). Moreover, activation of EP4 receptors attenuated the interaction of eosinophils with human pulmonary microvascular endothelial cells, with respect to adhesion under physiological flow conditions and transendothelial migration of eosinophils (Konya et al., 2011). EP4 agonist treatment inhibited cell surface clustering of β2 integrins and L-selectin shedding of eosinophils, which were all abolished using ONO AE3-208 (Konya et al., 2011). Interestingly, in a model of allergic airway inflammation, mice lacking EP4 had only slightly increased inflammatory cell infiltration in the lung compared to wild type mice, while EP3 knock-out mice developed much more pronounced allergic inflammation (Kunikata et al., 2005). This latter finding suggests that murine studies cannot be easily extrapolated to human pathobiology.

## 5 Airways

In the pulmonary environment, PGE_2_ attenuates inflammatory responses and reduces tissue injury of the airways (Vancheri et al., 2004). Additionally, COX-1 and -2 deficient mice show increased allergic responses in the airways (Gavett et al., 1999; Nakata et al., 2005). Consequently, selective COX-1 and -2 inhibitors enhanced allergic inflammation and airway hyperresponsiveness (Peebles et al., 2002). PGE_2_ was also shown to play a bronchoprotective role in patients with asthma (Pavord et al., 1993; Melillo et al., 1994; Aggarwal et al., 2010). Similarly, intratracheal administration of PGE_2_ diminished the ovalbumin-induced early and late phase airway responses in rats (Martin et al., 2002). Isolated tracheal and bronchial preparations from mice, rats, guinea pigs, monkeys and humans were all relaxed by PGE_2_, and this effect was mediated by the EP4 receptor only in human and rat airways, but by the EP2 receptor in the other species (Buckley et al., 2011; Benyahia et al., 2012). These important observations — EP4 receptor-mediated inhibitory effect on eosinophil effector functions and direct bronchodilator action — might contribute to the development of next generation bronchodilators as an alternative to the widely used β_2_ adrenoceptor agonists, which fail to treat the underlying tissue inflammation.

Consistent with its bronchoprotective action, PGE_2_ inhibited bronchial smooth muscle cell proliferation and migration through EP4 (Sastre et al., 2011; Aso et al., 2013). Moreover, EP4 receptor activation caused apoptosis in human adult lung fibroblasts by reducing the expression of survivin, an inhibitor of apoptosis and attenuating Akt activity (Huang et al., 2009), and partially mediated the inhibitory effect of PGE_2_ on the migration of human fetal fibroblasts in a cAMP/PKA/PI3K-dependent manner (Y.J. Li et al., 2011). Therefore, EP4 agonists might be a useful therapeutic intervention for airway inflammatory diseases such as asthma and chronic bronchitis due to their bronchoprotective, antifibrotic and anti-inflammatory effects. In contrast, blockade of EP4 receptors reversed the senescence of lung fibroblasts from COPD patients, suggesting a potential usefulness of EP4 receptor antagonists in COPD (Dagouassat et al., 2013).

## 6 Gastrointestinal tract

It is long known that NSAIDs contribute to worsening of gastrointestinal mucosal damage and inflammation in humans, and are a major risk factor for ulcer disease and gastrointestinal hemorrhage (Kaufmann & Taubin, 1987; Bjarnason et al., 1993; Felder et al., 2000; Laine et al., 2008). Experimental gastric damage as induced by the COX inhibitor indomethacin or by a mixture of ethanol plus HCl in mice and rats are ameliorated by PGE_2_ and an EP1 agonist (Suzuki et al., 2001; Takeuchi et al., 2001), while duodenal and intestinal lesions are prevented by EP3 and EP4 agonists (Kunikata et al., 2001; Kunikata et al., 2002). Part of the EP4 receptor-mediated intestinal mucosal protection might be due to stimulation of duodenal bicarbonate and mucus secretion (Takeuchi et al., 1997; Araki et al., 2000; Aihara et al., 2007), its anti-apoptotic effect on epithelial cells (Hoshino et al., 2003), vasodilation (Hattori et al., 2008) and vascular endothelial growth factor (VEGF) release to promote angiogenesis and mucosal healing (Hatazawa et al., 2007).

Consistent with the anti-inflammatory action of prostaglandins in the gut, COX-1 and -2 deficient mice show augmented colitis induced experimentally by oral administration of dextran sodium sulfate (DSS) (Morteau et al., 2000). The involvement of EP receptors was addressed by inducing colitis with 3% DSS in mice deficient for DP, EP1, EP2, EP3, FP, IP or TP receptor (Kabashima et al., 2002). Only EP4 receptor deficient mice developed severe colitis, similar to mice treated with the EP4-selective antagonist ONO AE3-208. Switching off the EP4 receptor abolished the mucosal barrier function and led to edema formation, damage of the epithelial layer and infiltration of immune cells, e.g. neutrophils and CD4^+^ T cells in the colon. Two different EP4-selective agonists, ONO AE1-734 and AGN205203, reversed the pathology of DSS colitis in wild-type mice, an effect that was strongly suppressed by an EP4 antagonist. On the cellular level, EP4 receptor stimulation protected against colon epithelial apoptosis, prevented goblet cell depletion, and promoted the regeneration of the epithelial layer. A substantial observation was the upregulation of EP4 mRNA following DSS-induced colitis in mice and rats (Kabashima et al., 2002; Nitta et al., 2002; Jiang et al., 2007).

These results were at variance with a study that addressed the role of EP4 receptors in a different, more severe, model of colitis induced by trinitrobenzene sulfonic acid. EP4 receptor stimulation by PGE_1_-OH or misoprostol, a non-selective EP receptor agonist, aggravated colitis and reduced survival rate, which was attributed to EP4 receptor-mediated IL-23 secretion from dendritic cells leading to accumulation of IL-17-producing cells in the colon (Sheibanie et al., 2007). These seemingly contradicting findings of EP4 receptors being beneficial in one colitis model, but deleterious in the other more severe model, might be due to the inhibitory role of EP4 receptors in fibroblast migration, thereby hampering wound healing and tissue repair (Rieder et al., 2010).

A clinical pilot study recently addressed the protective effect of the EP4 receptor agonist ONO-4819CD, a prodrug, in a small group of patients (four patients in the active treatment group, three in the placebo group) suffering from mild-to-moderate ulcerative colitis (Nakase et al., 2010). A tendency towards improvement of clinical symptoms as well as histological scores was noted in EP4 agonist-treated patients.

Disruption of the intestinal mucosal barrier is characteristic in several gastrointestinal diseases including ischemia–reperfusion, inflammatory bowel disease and NSAID-induced gastropathy (DeMeo et al., 2002). PGE_2_ was shown to induce gastrointestinal anion secretion as a mechanism of mucosal barrier protection (Bunce & Spraggs, 1990). PGE_2_ stimulated chloride secretion in ischemia-injured porcine ileal mucosa, which was followed by significant increase in transepithelial electrical resistance referring to recovery of epithelial barrier function (Moeser et al., 2004). The chloride channel ClC-2 was identified as the principal path for this response and showed co-expression with occludin in epithelial tight junctions (Moeser et al., 2004). Lubiprostone, a PGE_1_ derivative, was also shown to enhance gastrointestinal mucosal barrier function and was characterized as a selective activator of ClC-2, acting very similar to PGE_2_ (Moeser et al., 2007). Lubiprostone is currently approved for the treatment of chronic constipation in the U.S.A., Switzerland, United Kingdom and Japan. In rat and human gut preparations lubiprostone was found to interact with EP1 and EP4 receptors (Bassil et al., 2008) and to increase the expression of ClC-2 and cystic fibrosis transmembrane conductance regulator (CFTR). Moreover, lubiprostone induced contraction of villi and proximal colonic plicae and increased mucus exocytosis in goblet cells (Jakab et al., 2012). Similar to its gastrointestinal effects, lubiprostone was shown to stimulate tracheal submucosal gland secretion in pigs, sheep and humans without producing bronchoconstriction (Joo et al., 2009). An EP4-selective antagonist (L-161982) prevented the lubiprostone-induced chloride transport and submucosal gland secretion in sheep airways, and an EP4 agonist (L-902688) mimicked the effects of lubiprostone (Cuthbert, 2011). In a human airway epithelial cell line, EP4 receptor stimulation increased chloride efflux through CFTR (Joy & Cowley, 2008). Consequently, lubiprostone and other EP4 agonists could find therapeutic application in diseases with reduced airway fluid secretion.

## 7 Vasculature

The EP4 receptor has been first identified owing to its role in the PGE_2_-induced relaxation of the piglet saphenous vein (Coleman et al., 1994). In this first report, the EP1, EP2 and EP3 receptors were ruled out as being responsible for the vasodilator effect of PGE_2_ and, thus, the existence of a fourth EP receptor was proposed. Today, it is generally accepted that activation of EP1 and EP3 receptor subtypes induces vasoconstriction, whereas the EP2 and EP4 receptors expressed on smooth muscle cells mediate vasodilation (Norel, 2007). The EP4 receptor was further established as an important regulator of vascular tone in isolated perfused rat kidneys where the PGE_2_-induced relaxation of renal afferent arterioles was EP4 receptor-dependent (Tang et al., 2000). Coupling to G_αs_ and the subsequent formation of cAMP determined the observed vasorelaxation. A different signaling pathway was proposed to mediate the vasorelaxing effect of EP4 receptors in aortic rings of mice where increased eNOS activity and intracellular cGMP accumulation were found (Hristovska et al., 2007). The PGE_2_-induced relaxation was mimicked by an EP4 agonist (ONO AE1-329), and reversed by an EP4 receptor antagonist (ONO AE3-208). Consistent with these findings, relaxation was attenuated significantly in aortic rings from EP4^−^/^−^ mice but remained unaffected in EP2^−^/^−^ mice. Moreover, the in vivo hypotensive response to PGE_2_ was attenuated in eNOS-deficient mice (Hristovska et al., 2007). EP4-selective agonists also induced relaxation of the human pulmonary vein, an effect that was prevented by blockade of EP4 receptors. In contrast, the pulmonary artery was not relaxed after EP4 receptor activation. Accordingly, smooth muscle cells of pulmonary vein express more EP4 mRNA than cells isolated from pulmonary artery (Foudi et al., 2008).

### 7.1 Ductus arteriosus

EP4 deficiency in mice is perinatally lethal. Ninety five percent of animals were found to die due to patent ductus arteriosus, the fetal vessel that bridges the pulmonary and systemic circulation until birth (Segi et al., 1998). The remaining 5% survived and lived on for a year with partially closed DA. The patency of ductus arteriosus during the fetal period was presumed to be maintained principally by the dilator effect of the EP4 receptor, while its closure was thought to be induced by immediate withdrawal of the dilator prostaglandin PGE_2_ as well as active contraction exerted by increased oxygen tension (Smith, 1998). Later studies in rats revealed that EP4 receptor-mediated signals promote ductus arteriosus closure by hyaluronic acid accumulation to form an intimal cushion at birth, by stimulating the migration and hyaluronic acid production of smooth muscle cells (Yokoyama et al., 2006). The paradox of patent ductus arteriosus is that vasodilator EP4 receptors are needed for maintaining patency of the ductus in fetal life; however, EP4 receptor expression decreases before birth but still induces intimal cushion formation which leads to direct closure of the ductus. Thus, ductus arteriosus closure is not merely due to vasoconstriction as previously believed (Ivey & Srivastava, 2006). In rodents, ductus arteriosus closure occurs within hours after birth, while in humans, due to its larger diameter, closure of the ductus requires highly developed intimal cushion formation which takes several days to be completed (Gentile et al., 1981; Tada & Kishimoto, 1990). Therefore, studies in larger mammals are required to clarify whether EP4 agonists are directly applicable to neonatal medicine to improve the outcomes of low birth-weight infants who suffer from patent ductus arteriosus.

### 7.2 Ischemia

The potential beneficial roles of EP receptors in cardiovascular function have been reviewed recently (J.-i. Suzuki et al., 2011; Tang et al., 2012). Endogenous PGE_2_, by stimulating EP4 receptors was found to be cardioprotective in a murine cardiac ischemia/reperfusion model (Xiao et al., 2004). EP4^−/−^ mice showed increased cardiac infarct size as compared to wild-type mice. Among EP receptors, the EP4 receptor was shown to be highly expressed in the mouse heart and exclusively mediated the protective effects of PGE_2_. The EP4 receptor agonists, ONO AE1-329 and ONO-4819-CD (300 μg/kg subcutaneously), a prodrug for in vivo application that is converted to an active metabolite in the circulation (Maruyama et al., 2002), were cardioprotective via activating cAMP (Xiao et al., 2004). However, the mechanisms of how elevation of intracellular cAMP levels exerts cardioprotection in ischemia has not yet been elucidated yet, but might reflect EP4 receptor-mediated vasodilator or platelet inhibitory responses as described below (Philipose et al., 2010). An additional important aspect of the EP4 receptor-mediated cardioprotective function was reported in rats, where EP4RAG, an EP4-selective agonist, prevented myocardial dysfunction after infarction (Hishikari et al., 2009). These experiments revealed that EP4 receptor activation leads to suppression of inflammatory cytokines, including TNF-α, IL-1β, IL-6, and MCP-1, thereby limiting the infiltration of macrophages at sites of ischemia/reperfusion. Furthermore, EP4RAG attenuated the activity of matrix metalloprotease-2 and -9 in the tissue, and inhibited MCP-1-induced migration of monocytes in vitro, which might have added to the cardioprotective action of the EP4 receptor (Hishikari et al., 2009). The anti-inflammatory potential of the EP4 receptor in the heart was further demonstrated by the same group in models of murine heart transplantation and rat autoimmune myocarditis (Ogawa et al., 2009; Ngoc et al., 2011).

PGE_2_ has also been described to protect against liver injury caused by ischemia/reperfusion, chemotoxicity and bacterial infection (Stachura et al., 1981; Miyazaki et al., 1983; Takano et al., 1998). These protective effects were hypothetically explained by increased liver perfusion, anti-platelet effect, inhibition of cytokine release and direct cytoprotective function of PGE_2_ (Masaki et al., 1992). Out of the four EP receptors only EP4 receptors were found to be upregulated on hepatocytes in an ischemic mouse model (Kuzumoto et al., 2005). Activation of EP4 receptors by the EP4-selective agonist ONO AE1-329 provided strongest protection against hepatic injury as compared to other selective EP receptor agonists. In detail, the EP4 agonist reduced the serum levels of hepatic injury markers and inhibited the release of proinflammatory cytokines such as TNF-α and IFN-γ, and the chemokines MCP-1 and IP-10, and also decreased the expression of E-selectin and ICAM-1 adhesion molecules in the ischemic liver (Kuzumoto et al., 2005). Importantly, neutrophil infiltration into the liver was also found to be attenuated after EP4 receptor stimulation. The high therapeutic potential of EP4 receptors in the liver was unequivocally indicated by the observation that 80% of lethally injured mice treated with an EP4 agonist were alive two days after reperfusion, while only 14% of vehicle-treated mice survived (Kuzumoto et al., 2005).

Prostaglandins play controversial roles in neurological disorders as activation of COX, particularly COX-2, has been shown to worsen cerebral injury in several animal models (Hewett et al., 2006). At the same time, patients taking COX-2 inhibitors are at increased cerebrovascular and cardiovascular risk, because COX-2 inhibition selectively removes the inhibitory effect of vascular PGI_2_ synthesis on platelet activity without attenuating COX-1-derived thromboxane synthesis in platelets (Funk & FitzGerald, 2007). Supporting the beneficial effects of COX metabolites, the EP4 receptor was reported to play a protective role in a mouse model of cerebral ischemia (Liang et al., 2011). Treatment of mice after ischemia with ONO AE1-329 reduced infarct size and prevented long-term locomotor deficits. EP4 receptors were found to abound on neurons and became markedly upregulated in endothelial cells after ischemia/reperfusion, suggesting that the dual EP4 receptor signaling of neurons and endothelial cells imparts cerebroprotection. Conditional deletion of neuronal or endothelial EP4 receptors aggravated the cerebral deficits and decreased cerebrovascular reperfusion in the same study. Increased levels of activated eNOS in cerebral microvessels following EP4 agonist treatment were proposed to underlie the cerebral protective effect of the EP4 agonist. The inhibitory effects of EP4 receptor activation on macrophages and the implications of EP4 receptors in vascular inflammation and atherosclerosis are discussed above.

### 7.3 Platelet aggregation

An important prerequisite of cardiovascular homeostasis is tight regulation of platelet aggregation. We recently observed that stimulation of EP4 receptors on human platelets inhibits platelet aggregation and in vitro thrombus formation of human whole blood (Philipose et al., 2009, 2010). Similar observations were reported in parallel by three other groups (Iyu et al., 2010; Kuriyama et al., 2010; Smith et al., 2010). The EP4 agonist ONO AE1-329 attenuated the pro-aggregatory effect of ADP with an IC_50_ value of 30 nmol/L, that of platelet activating factor with an IC_50_ value of ~10 nmol/L and those of collagen and the thromboxane mimetic U46619 with an IC_50_ value of 3 nmol/L. Two different EP4 antagonists completely reversed the inhibitory effect of ONO AE1-329 on platelet aggregation, and rendered PGE_2_ to being a potent pro-aggregatory mediator. The anti-aggregatory effect of the EP4 agonist on stimulated platelets was characterized by reduced intracellular Ca^2+^ mobilization and phosphorylation of vasodilator-stimulated phosphoprotein (VASP), inhibition of glycoprotein IIb/IIIa activation, and downregulation of P-selectin (Iyu et al., 2010; Kuriyama et al., 2010; Philipose et al., 2010). Surprisingly, ONO AE1-329 was at least 30-times less potent in inhibiting the aggregation of rat, guinea pig and mouse platelets than that of human platelets (Kuriyama et al., 2010; Philipose et al., 2010). Moreover, the anti-aggregatory response to PGE_2_ in mice was observed to be mediated by the IP receptor rather than the EP4 receptor as revealed by IP- and EP4-deficient mice, respectively (Fabre et al., 2001; Kuriyama et al., 2010). These findings shed light on the previously described dual effect of PGE_2_ on platelet aggregation, namely that at lower concentration PGE_2_ potentiated, while at higher concentration inhibited, platelet aggregation (Salzman et al., 1972; Shio et al., 1972; Weiss et al., 1976; Thierauch & Prior, 1991; Vezza et al., 1993). From these studies it may be inferred that the pro-aggregatory effect of PGE_2_ in human platelets is mediated by EP3 receptors, while EP4 receptors are responsible for its anti-aggregatory properties (Matthews & Jones, 1993; Mao et al., 1996; Ma et al., 2001; Gross et al., 2007; Heptinstall et al., 2008).

### 7.4 Endothelial function

**Fig. 3:**
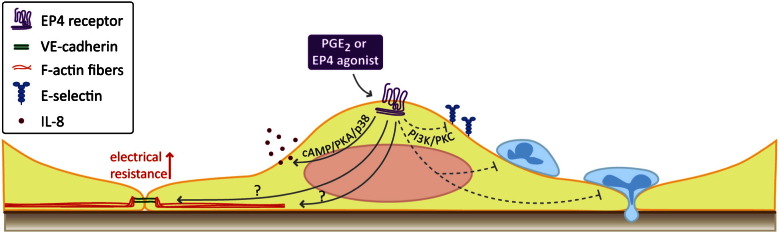
The EP4 receptor promotes endothelial barrier function. EP4 receptor activation induces rapid cortical actin polymerization and VE-cadherin expression in the endothelial junctions, which results in markedly increased electrical resistance of the cell monolayer. Furthermore, E-selectin upregulation, and leukocyte adhesion and transendothelial migration as induced by TNF-α treatment is prevented by EP4 receptor activation. EP4 receptor-mediated E-selectin down-regulation is PI3K/PKC-dependent; however, none of the generally used EP4 receptor signaling modules (cAMP, eNOS, Rac1, PI3K, p38, ERK1/2) are involved in the barrier-enhancing effect of EP4 receptor. In contrast, the cAMP/PKA/p38 MAP kinase pathway mediates the EP4 receptor-stimulated release of IL-8 in endothelial cells.

PGE_2_ along with PGI_2_ has been shown to increase human pulmonary artery endothelial barrier function. Particularly, PGE_2_ was shown to reduce dextran permeability of resting and thrombin-stimulated human pulmonary artery endothelial cells and to enhance endothelial electrical resistance via cAMP/PKA/Rap1/Rac signaling (Birukova et al., 2007). In a recent study we have identified the EP4 receptor as being responsible for the enhanced endothelial barrier function in response to PGE_2_ (Konya et al., 2013). Using human pulmonary microvascular endothelial cells as a model system, we observed that only an EP4 receptor agonist (ONO AE1-329), but not EP1, EP2 and EP3 agonists mimicked the barrier enhancing effect of PGE_2_ (Fig. 3). Similar responses were recorded in human coronary artery endothelial cells. The barrier enhancement was totally abolished by an EP4 antagonist (ONO AE3-208) and by preventing actin polymerization. On the cellular level PGE_2_ and the EP4 agonist induced cortical actin polymerization and enhanced endothelial junctional expression of VE-cadherin (Fig. 3). Interestingly, the classical cAMP/PKA signaling module, eNOS or Rac1, PI3K or p38 kinases were not involved in this system. Additionally, EP4 receptor activation facilitated wound healing of pulmonary microvascular endothelial monolayers. Endothelial E-selectin upregulation, neutrophil adhesion and transmigration, induced by TNF-α pretreatment of endothelial cells, were also inhibited by endothelial EP4 receptor stimulation. The reduction of E-selectin expression depended on PI3K/PKC kinases. However, the exact mechanism of EP4 receptor-mediated endothelial barrier function awaits further investigation. Taken together, these observations indicate that EP4 agonists offer a potential novel approach to diseases with increased vascular permeability and neutrophil extravasation (Birukova et al., 2007; Konya et al., 2013).

By contrast, another recent report suggested that human pulmonary microvascular endothelial cells show enhanced release of the chemokine IL-8 upon stimulation with PGE_2_ or ONO AE1-329 for 24 h (Aso et al., 2012). This pro-inflammatory action of EP4 receptors was mediated by cAMP/PKA and p38 MAP kinase but without involving ERK1/2 (Fig. 3). Whether the EP4 receptor-induced IL-8 release from endothelial cells actually results in neutrophil recruitment to the tissue has not yet been addressed, but similar EP4 receptor-mediated expression of proinflammatory cytokines was observed in airway and colon epithelial cell lines (Dey et al., 2009; T. Li et al., 2011). On a similar line, EP4 receptor activation in murine cerebrovascular endothelial cells upregulated intercellular adhesion molecule-1 (ICAM-1) expression (Park et al., 2013).

### 7.5 Angiogenesis

Beside its vasoprotective effects, PGE_2_ can induce angiogenesis of endothelial cells (Salcedo et al., 2003). Further studies demonstrated that PGE_2_ and EP4 receptor-selective agonists (PGE_1_-OH and ONO-AE1-329) induced migration and tubulogenesis of pulmonary microvascular endothelial cells derived from control mice, while they had no effect on EP4 deficient endothelial cells (Rao et al., 2007). The EP4 agonist did not induce cell proliferation but stimulated endothelial cell migration, an effect that depended on ERK, but was independent of the classical cAMP pathway. Additionally, the same EP4 agonists promoted angiogenesis in vivo (Rao et al., 2007; Zhang & Daaka, 2011). Likewise, inflammatory lymphangiogenesis promoting the formation of granulation tissue in matrigel plugs was abrogated in EP3- and EP4-deficient mice (Hosono et al., 2011).

In human umbilical vein endothelial cells (HUVEC) PGE_2_ induced angiogenesis by activating the NO/cGMP signaling pathway via PKA/PI3K/Akt-dependent elevation of eNOS activity (Namkoong et al., 2005). Activation of EP4 receptors by PGE_2_ promoted in vitro tube formation in human neonatal dermal microvascular endothelial cells, ex vivo vessel outgrowth of murine aortic rings, and in vivo angiogenesis in sponge implants in mice (Zhang & Daaka, 2011). The selective involvement of EP4 receptors was proved by EP4-selective agonists and antagonists, and siRNA knock-down of the receptor. EP4 receptor activation in endothelial cells caused intracellular cAMP formation. In agreement with this observation, elevation of intracellular cAMP by forskolin has previously been reported to stimulate angiogenesis via PKA-mediated VEGF expression and Epac-dependent PI3K/Akt/eNOS signaling (Namkoong et al., 2009). Further signaling pathways in the pro-angiogenic effect of EP4 stimulation were elucidated to include EP4 receptor coupling to Gαs, activation of PKA catalytic subunit γ, and activation of Rap1A, HSPB6 and eNOS, i.e. known phosphorylation targets of PKA that have been shown to mediate tube formation (Zhang & Daaka, 2011). These findings clearly demonstrate that EP4 receptors mediate PGE_2_-induced angiogenesis in mouse and human endothelial cells. Interestingly, this occurs in mice in an ERK-associated but PKA-independent manner, whereas it depends on cAMP/PKA/eNOS/cGMP pathways in human endothelial cells.

Collectively, a large body of evidence suggests that EP4 agonists might be promising novel approaches to control vascular disease due to the broad biological functions of the EP4 receptor in vasculature. These include anti-ischemic, vasodilator, and platelet inhibitory actions, stimulation of endothelial barrier function and angiogenesis, complemented by anti-inflammatory properties.

## 8 Kidney

PGE_2_ plays diverse and important roles in the regulation of renal function (Hao & Breyer, 2008). Accordingly, the kidney is a major target of adverse effects of COX inhibitors particularly in the elderly, which include salt retention, hypertension, and blunted renal function (Harirforoosh & Jamali, 2009). Among the EP receptors, EP4 receptor (and to some extent EP2) is key to renal homeostasis by regulating cell proliferation, vascular tone and renin secretion. Cultured juxtaglomerular cells show pronounced renin secretion in response to PGE_2_ and EP4 agonists (Friis et al., 2005). This response appears to be mediated by cAMP-dependent hyperpolarization and inhibition of Ca^2+^ currents that paradoxically inhibit renin-containing granule exocytosis. In vivo, low salt diet stimulates kidney renin mRNA levels and plasma renin concentrations in wild-type mice but not in EP4 knock-out mice or in mice treated with the EP4 antagonist ONO-AE3-208 (Poschke et al., 2012). Similar results were obtained in mice treated with furosemide (Nusing et al., 2005; Facemire et al., 2011). Vascular tone in the kidney also depends on EP4 receptors: the PGE_2_-induced vasodilatation at low concentrations is abolished, while EP1-mediated vasoconstriction at higher concentrations of PGE_2_ is augmented, in EP4-deficient mice (Purdy & Arendshorst, 2000; Schweda et al., 2004).

PGE_2_ and EP receptors including EP4 play important roles in kidney development, as suggested by a reduced glomerular size in EP4-deficient mice (Frolich et al., 2012). In vitro, PGE_2_ acting via EP4 receptors augmented the survival of glomerular epithelial cells deprived of serum (Aoudjit et al., 2006), and an EP4 receptor agonist stimulated proliferation and decreased apoptosis of renal epithelial cells (Yamamoto et al., 2010). PGE_2_ and EP4 receptors have been suggested to be involved in autosomal dominant polycystic kidney disease, as they induce proliferation and chloride secretion in polycystin-1 deficient epithelial cells, while inhibiting proliferation in polycystin-1 expressing cells (Liu et al., 2012).

Several other pathologies of the kidney are counterbalanced by the PGE_2_/EP4 axis, suggesting that EP4 agonists might be a useful therapeutic option in renal disease. In a mouse model of renal diabetes insipidus, EP4 receptor stimulation with ONO AE1-329 compensated the loss of vasopressin V2 receptors probably by activating aquaporin-2 in tubule epithelial cells (Li et al., 2009; Olesen et al., 2011). The same EP4 receptor agonist protected against anti-glomerular basement membrane antibody-associated nephritis in mice (Nagamatsu et al., 2006). In a murine model of ureteral obstruction, the development of renal fibrosis, the accumulation of macrophages, and the formation of proinflammatory and profibrotic cytokines were significantly augmented in the kidneys of EP4 knock-out mice and suppressed by an EP4 agonist, ONO-4819 (Nakagawa et al., 2012). In a mercury chloride-induced rat model of acute kidney failure, a systemically administered EP4 agonist (CP-044,519-02) reduced serum creatinine levels and increased the survival rate of the animals (Vukicevic et al., 2006). Similarly, treatment with the EP4 agonist delayed the progression of chronic renal failure as induced by 5/6 nephrectomy (Vukicevic et al., 2006).

In contrast, podocyte-specific overexpression of EP4 receptors significantly aggravated, while deletion of EP4 receptors in podocytes ameliorated, the decline of renal function in 5/6 nephrectomized mice (Stitt-Cavanagh et al., 2010). Interestingly, there is a positive feedback in PGE_2_ secretion, as COX-2 expression is induced in podocytes following EP4 stimulation through a PKA-independent mechanism (Faour et al., 2008), and COX-2 and EP4 receptors are found upregulated in compromised or regenerating tubule epithelial cells (Yamamoto et al., 2010; Nakagawa et al., 2012). EP4 receptor expression in glomeruli is increased following salt deprivation as are PGE_2_-evoked cAMP and renin responses in juxtaglomerular cells (Jensen et al., 1999). The EP4 receptor-induced stimulation of sodium absorption prompted by an activated renin/angiotensin II/aldosterone system is counterbalanced by the natriuretic action of EP1 receptors and diuretic action of EP3 receptors in the collecting duct (Fleming et al., 1998; Guan et al., 1998; Tamma et al., 2003).

## 9 Bone and cartilage

Osteoporosis is a bone degenerative disease of the elderly, induced by the imbalance of bone resorption by osteoclasts and bone formation by osteoblasts. Such loss of bone density increases the incidence of bone fractures. Among prostaglandins, PGE_2_ shows the most prominent activity in bone, being involved both in bone formation and resorption (Norrdin et al., 1990). The dual role of prostaglandins in bone dynamics is also reflected by epidemiological data showing that chronic use of COX-2 inhibitors is associated with reduced bone mineral density in males, while the opposite effect is observed in postmenopausal women not taking hormone replacement therapy (Richards et al., 2006). In clinical practice, COX inhibitors are being used to control ectopic bone formation, but their use in the treatment of fracture pain has raised concern with respect to a potential delay of fracture healing (Vuolteenaho et al., 2008).

Bone resorption is carried out by osteoclasts, which originate from bone marrow-derived mononuclear cells. EP1 and EP4 receptors were found to be highly expressed in a mouse osteoblastic cell line, and stimulation of EP4 receptors by 11-deoxy-PGE_1_, a purported EP4 receptor agonist, promoted osteoblast differentiation (Suda et al., 1996). PGE_2_ was found to induce in vitro osteoclast formation from bone marrow cells in the presence of primary murine osteoblasts, which was mimicked by 11-deoxy-PGE_1_. Additionally, osteoclast formation in response to PGE_2_, but also to TNFα, IL-1β or LPS, was ablated in cells isolated from EP4 deficient mice (Sakuma et al., 2000). In agreement with these data, an EP4 antagonist was found to attenuate LPS-induced osteoclast formation and bone destruction in rat periodontal tissue (Oka et al., 2012).

In contrast, osteoclast differentiation from human peripheral blood monocytes is blocked by EP4 receptor activation (Take et al., 2005). At the same time, EP4 receptors also seem to play a role in the bone anabolic effect of PGE_2_. COX-2 deficient mice presented with impaired healing of fractures, which was reversed by EP4 agonist treatment (Xie et al., 2009). EP4-deficient mice showed impaired bone formation in vivo in response to PGE_2_, while other EP receptor-deficient mice exhibited unchanged callus formation (Yoshida et al., 2002). The effect of PGE_2_ was mimicked by the EP4 agonist ONO AE1-329. Consistently, the EP4 agonist also induced the formation of mineralized nodules in bone marrow cultures from wild-type mice, but not EP4 deficient mice. Moreover, the same study reported that the EP4 agonist ONO 4819CD promoted de novo bone formation in experimental rat models of osteoporosis, induced by ovarectomy and immobilization, respectively (Yoshida et al., 2002). The protective effect of EP4 receptor stimulation against immobilization-induced bone loss is in line with the observation that mechanical strain applied to human osteoblasts down-regulates sclerostin, a potent inhibitor of bone formation, via COX-2 induction and EP4 receptor/ERK signaling (Galea et al., 2011).

Very recently, novel dual-action bone-targeting drug conjugates, consisting of EP4 receptor agonists and a bisphosphonate, alendronic acid, were reported (Arns et al., 2012). Radiolabeled conjugates of these drugs were administered to rats and were found to be taken up into the bone and released subsequently in a sustained manner, suggesting an attractive novel approach to modulating bone metabolism.

PGE_2_ and EP4 receptors may play a similar, dual role in cartilage homeostasis and arthritis. In cartilage of osteoarthritis patients PGE_2_ acting via EP4 receptors attenuated chondrocyte expression of connective tissue growth factor (Masuko et al., 2010), inhibited proteoglycan synthesis and promoted matrix degradation (Attur et al., 2008), while it also stimulated the release of osteoclast-activating factor RANKL (Moreno-Rubio et al., 2010).

Moreover, the EP4 receptor seems to be involved in inflammatory pain in experimental arthritis models and to promote Th1 differentiation, Th17 cell expansion, and IL-23 secretion by activated dendritic cells (Yao et al., 2009; Chen et al., 2010). Consequently, EP4 antagonists might have analgesic and anti-inflammatory effects in arthritis (Omote et al., 2002; Okumura et al., 2008; Colucci et al., 2010). Ultimately, EP4 knock-out mice were found to be resistant to type-II collagen antibody-induced arthritis (McCoy et al., 2002). In a positive feed-back loop, EP4 receptors are upregulated in arthritic tissue (Kurihara et al., 2001).

On the other hand, EP4 receptor activation in chondrocytes has been linked to PGE_2_-induced inhibition of matrix metalloprotease expression (Fushimi et al., 2007; Nishitani et al., 2010). In an ex vivo model of human pannus formation, i.e. aberrant proliferation of synovial tissue leading to joint destruction, endogenous PGE_2_ via activating its EP4 receptor, inhibited pannus growth and osteoclast activity (Shibata-Nozaki et al., 2011). These findings might be consistent with a crucial role of EP4 receptors in promoting acute inflammatory responses and pain in arthritis, but also with limiting cartilage and bone destruction in the chronic phase.

## 10 Cancer

The role of prostaglandins has extensively been studied in cancer, and COX-2 has emerged as a potential therapeutic target in some tumors. COX-2 expression has been found to correlate with cancer progression in experimental models and in many types of cancers, and pharmacological inhibition or genetic ablation of COX-2 reduces tumor cell proliferation and metastasis (Cebola & Peinado, 2012). Moreover, long-term administration of COX inhibitors such as acetylsalicylic acid can exert significant prophylactic effects against certain cancers (Harris, 2009). The significant adverse effects of COX inhibitors need to be considered, though, and might be avoided by selectively addressing the respective receptors for prostaglandins instead of non-selectively inhibiting prostaglandin production.

Revisiting the roles of different EP receptors in cancer development, a large body of evidence supports EP4 receptors to predominantly mediate the overall pro-tumorigenic action of PGE_2_. The potential involvement of EP4 receptors has been described in many different types of cancer. Activation of EP4 may confer diverse cellular responses, such as promoting angiogenesis, proliferation, motility and metastasis, or delaying apoptosis, of tumor cells. These notions are based on studies using selective EP4 antagonists and agonists as well as mice that have been made deficient in EP4 receptors. In agreement with these findings, EP4 receptors are expressed on sprouting angiogenic cells, immune cells and the tumor cells itself, which are activated by PGE_2_ in an autocrine or paracrine manner.

For instance, EP4 receptor expression was observed to increase during transition of colorectal adenoma to carcinoma (Chell et al., 2006) and EP4 receptor over-expression favored an anchorage-independent phenotype in otherwise anchorage-dependent human colorectal adenoma cells (Chell et al., 2006; Hawcroft et al., 2007). Consistently, the EP4 antagonist ONO AE3-208 attenuated colony forming capacity. PGE_2_ stimulates migration of colon cancer cells via EP4/β-arrestin 1/c-Src signaling that transactivates and phosphorylates the epidermal growth factor receptor (Buchanan et al., 2006). EP4 receptor over-expression resulted in the suppression of apoptosis in a cAMP/CREB-dependent manner, and enhanced anchorage-independent growth, of a human colon cancer cell line (Hawcroft et al., 2007). In a different colon carcinoma cell line EP4 receptor was found to drive in vivo growth by augmenting tumor vascularization, and delaying apoptosis (Pozzi et al., 2004), while colon carcinogenesis was inhibited in mice lacking EP4 but not EP2 receptors, and by the EP4 antagonist ONO AE2-227 (Mutoh et al., 2002). Interestingly, over-expression of k-Ras, an oncogene involved in malignant transformation of many tumors, facilitated COX-2 and EP4 receptor expression in colon carcinoma cells (Wu et al., 2010).

EP4 mRNA levels were also found elevated in cervical carcinoma compared with normal cervix (Sales et al., 2001), and expression of COX-2 and EP4 receptors in transitional cell carcinoma of the upper urinary tract was associated with metastasis and shorter survival of patients (Miyata et al., 2005). COX-2 derived PGE_2_ stimulated the migration and CCR7 expression of breast cancer cells via EP4 receptors linked to PKA and Akt signaling (Timoshenko et al., 2003; Pan et al., 2008). In a murine breast cancer model, EP4 receptors promoted lymphangiogenesis and ensuing metastasis (Xin et al., 2012). In breast cancer patients, expression of the EP4 receptor correlated with enhanced lymphatic invasion (Pan et al., 2008), and the EP4 antagonists, AH23848 and ONO AE3-208, attenuated lung metastasis of these tumor cells in mice, while ONO-AE2-227 prevented their osteolytic activity in vitro (Ohshiba et al., 2003; Ma et al., 2006). Similarly, EP4 receptor stimulation resulted in cAMP/EPAC-mediated activation of RAP small GTPase and augmented migration of renal carcinoma cells (Wu et al., 2011). Ovarian cancer cells responded to activation of PGE_2_/EP4 receptor signaling with VEGF expression and tumor cell invasion, by activating tumor-associated matrix metalloproteinases (Spinella et al., 2004). EP4 receptors facilitated pulmonary metastasis of lung carcinoma cells injected intravenously in mice and liver metastasis after intrasplenic injection of colon cancer cells (Yang et al., 2006). Treatment of mice with ONO AE3-208 or siRNA knock-down of EP4 receptor in the tumor cells ameliorated tumor growth and metastasis.

In mouse skin, overexpression of EP4 receptors enhanced non-melanoma skin tumorigenesis (Rundhaug et al., 2011), and PGE_2_ acting via EP4 receptors stimulated the migration of human melanoma cells (Singh et al., 2011). Consistently, the EP4 antagonist ONO AE3-208 prevented melanoma-induced bone metastasis and osteolysis in mice (Takita et al., 2007). Other types of cancer that might be sensitive to EP4 receptor stimulation include mycosis fungoides (Kopp et al., 2010), pancreatic adenocarcinoma (Funahashi et al., 2008), esophageal adenocarcinoma (Ogunwobi et al., 2006; Jimenez et al., 2010), endometrium cancer (Catalano et al., 2011), prostate cancer (Terada et al., 2010; Miao et al., 2012), neuroblastoma (Rasmuson et al., 2012), and glioblastoma (Kambe et al., 2009).

In addition to directly stimulating tumor cell proliferation and motility, EP4 receptor-mediated PGE_2_ signals can down-regulate immune responses, thereby hampering tumor-limiting mechanisms. In detail, tumor-derived PGE_2_ stimulating EP4 receptors curb natural killer cell and γδ T cell activity (Kundu et al., 2009; Martinet et al., 2010; Holt et al., 2011), and cytokine secretion from macrophages (Qian et al., 2011) and microglia (Nakano et al., 2008). Dendritic cell maturation is blocked and their differentiation is diverted towards a phenotype referred to as myeloid-derived suppressor cells (Obermajer et al., 2011a, 2011b). Importantly, in a positive feedback loop PGE_2_ induced further COX-2 leading to exaggerated PGE_2_ biosynthesis in immature myeloid cells (Ogunwobi et al., 2006; Obermajer et al., 2011a). Similarly, PGE_2_/EP4 receptor-mediated induction of Foxp3/CD4/CD25^+^ T regulatory cells was shown to result in enhanced tumor burden in mice injected with lung cancer cells (Sharma et al., 2005). Conversely, PGE_2_ supported the recruitment of tumor-promoting macrophages to mouse gastric tumors thereby enhancing tumor growth (Oshima et al., 2011).

Taken together, several studies have demonstrated that EP4 antagonists might have beneficial effects on carcinogenesis and tumor progression. Recent studies, however, suggest that the EP4 receptor might also act as tumor suppressor, for instance in human B cells (Murn et al., 2008). The EP4 receptor provides a negative feedback signal to proliferation in response to B cell receptor activation, suppressing growth in a PGE_2_-dependent manner. B cell receptor activation upregulates EP4 receptors on immature B cells and promotes their apoptosis (Prijatelj et al., 2011). In B cell lymphomas, EP4 receptors are down-regulated and growth suppression is diminished (Murn et al., 2008). In another study, activation of EP4 receptors inhibited the growth of four different human gastric carcinoma cell lines (Okuyama et al., 2002).

## 11 Miscellaneous

In addition to what is described above, EP4 receptors may be useful therapeutic targets in various other organs, including eye, inner ear, brain or female reproductive tract. Topical application of the EP4 agonist prodrug PF-04475270 lowered ocular pressure through its active metabolite CP-734432 in normotensive dogs (Prasanna et al., 2009). However, conjunctival hyperemia and corneal neovascularization were also observed (Aguirre et al., 2009). EP4 knock-out mice exhibit a slight hearing deficit at baseline and are more susceptible to acoustic trauma as reflected by increased loss of outer hair cells. An EP4 antagonist recapitulated these effects while an EP4 agonist prevented hearing loss in wild-type mice (Hamaguchi et al., 2012). EP2 and EP4 agonists stimulated the production of VEGF in the spiral ganglion suggesting a beneficial effect in diseases like acute sensorineural hearing loss (Hori et al., 2010).

EP4 receptors are also involved in mediating various effects exerted by PGE_2_ in female sexual organs. Blockade of EP4 receptors inhibited the proliferation and invasion of human endometriotic cells, and induces their apoptosis (Banu et al., 2009; Lee et al., 2011). PGE_2_ stimulated follicle maturation in immature rat ovaries through upregulation of IL-8 and this was mimicked by an EP4 agonist (El-Nefiawy et al., 2005). An EP4 agonist also stimulated ovulation in mice (Xiao et al., 2008). In contrast, PGE_2_ delayed nuclear maturation and fertilization of mouse and monkey oocytes, probably through EP2 and/or EP4 receptors (Duffy et al., 2010). Endogenous PGE_2_ mediated LPS-induced cervical ripening in rabbits, which was reversed by an EP4 antagonist (Fukuda et al., 2007). An EP4 antagonist likewise prevented bladder hyperactivity induced by cyclophosphamide or PGE_2_ in rats (Chuang et al., 2012).

Finally, EP4 receptors are capable of modulating central nervous activity. EP4 receptor activation in a murine model of Alzheimer's disease, EP4 deficiency or the EP4 antagonist ONO-AE3-208 decreased amyloid-β levels in the brain and improved the behavioral performance of the animals (Hoshino et al., 2012). By contrast, intracerebroventricular administration of PGE_2_ or selective EP1 or EP4 agonists to mice caused anxiolytic-like effects in the elevated plus-maze and open field test, which was not observed in EP4-deficient animals, but could be reversed after blockade of serotonin 5-HT1A, dopamine D1 and GABA_A_ receptors (C. Suzuki et al., 2011). In a similar fashion, intracerebroventricular administration of PGE_2_ and the EP4 agonist, ONO-AE1-329, suppressed food intake in mice, and this effect was prevented by EP4 antagonism. Accordingly, the EP4 agonist also delayed gastric emptying, but elevated blood glucose levels (Ohinata et al., 2006). EP4 receptors are expressed in enteroendocrine cells and stimulate the release of incretins, such as glucagon-like peptide-1, in vitro and in vivo in mice (Coskun et al., 2013). A potential role of EP4 receptors in metabolic disease is further supported by enhanced adipogenesis from mouse embryonic fibroblasts of EP4-deficient mice and by the inhibitory effect of PGE_2_ acting via EP4 receptors and peroxisome proliferator-activated receptor-γ (Inazumi et al., 2011).

## 12 Conclusion

**Fig. 4:**
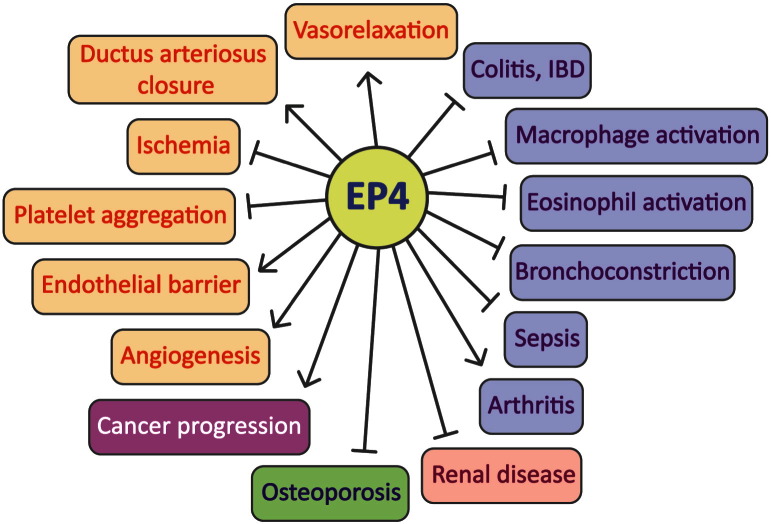
The effect of EP4 receptor activation in different diseases and cellular processes. Promotion of a respective response is shown by  while  indicates inhibitory modulation.

EP4 receptors are the most widely expressed PGE_2_ receptors in the body, and research over the past ten years or so has unraveled that numerous novel, or well-recognized biological effects of PGE_2_ can be attributed to EP4 receptor activation (Fig. 4). Among these are several anti-inflammatory roles, modulation of tissue development and regeneration, and regulation of vascular tone and hemostasis. In contrast to these EP4 receptor-mediated, assumingly beneficial actions of PGE_2_, pathogenic roles of EP4 receptors have been implicated in cancer growth and metastasis, and in some inflammatory diseases. As such, both EP4 receptor agonists and antagonists might become useful novel drug classes. Accordingly, the pharmaceutical industry has launched numerous EP4 drug development programs, and a variety of EP4 receptor agonists and antagonists have become available for experimental studies and further clinical development. While systemic administration of EP4 receptor antagonists might be well tolerated, agonists are better administered locally, thereby avoiding systemic side effects. Moreover, in consideration of the sometimes redundant biological roles of EP2 and EP4 receptors, dual EP2/EP4 antagonists might be of advantage. Finally, ligand-biased selectivity of EP4 receptor signaling may exist for G_αs_, G_αi_ and β-arrestin-dependent pathways and should be taken into consideration when interpreting biological effects of different EP4 ligands.

## Conflict of interest

The authors declare to have no conflict of interest.

## Figures and Tables

**Table 1:** EP4 receptor-selective agonists.

Name	Dose, concentration, affinity	Model, species	References
AGN205203	Ki = 81 nM 3 mg/kg	HEK-EP4, human Colitis, mouse	Jiang et al., 2007
APS-999 Na	50 μg/animal	Ovarian follicle growth, rat	El-Nefiawy et al., 2005
Cay10598 (19a)	Ki = 1.2 nM	HEK-EP4, human	Billot et al., 2003
CP-044,519-02	10 mg/kg/day	Acute and chronic kidney failure, rat	Vukicevic et al., 2006
EP4RAG	1–3 mg/kg	Myocardial dysfunction, rat	Hishikari et al., 2009
	10–50–100 nM	THP-1 monocyte migration	Hishikari et al., 2009
L-902688	10 nM–10 μM	Bronchi, human	Benyahia et al., 2012
	10 nM–10 μM	Pulmonary vein, human	Foudi et al., (2008)
Lubiprostone	50–500 nM	Short circuit current in tracheal epithelium and submucosal gland secretion, sheep	Cuthbert, 2011
ONO-4819CD	36 ng/kg	Ulcerative colitis, human	Nakase et al., 2010
	300 μg/kg	Cardiac ischemia, mouse	Maruyama et al., 2002
	1–30 μg/kg 3× a day	Inhibition of bone loss, de novo bone formation, rat	Yoshida et al., 2002
ONO AE1-329	25–100 μg/kg	Colitis, mouse	Nitta et al., 2002
	10 nM–10 μM	Bronchi, human	Benyahia et al., 2012
	30 nM	Eosinophil inhibition, human	Konya et al., 2011 and Luschnig-Schratl et al., 2011
	100 nM	Aortic rings, mouse	Hristovska et al., 2007
	10 nM–10 μM	Pulmonary vein, human	Foudi et al., 2008
	1 μM	Ductus arteriosus smooth muscle cells, rat	Maruyama et al., 2002 and Yokoyama et al., 2006
	300 μg/kg	Cardiac ischemia, mouse	Maruyama et al., 2002
	30–100 μg/kg	Hepatic ischemia, mouse	Kuzumoto et al., 2005
	30–300 μg/kg	Cerebral ischemia, mouse	Liang et al., 2011
	30 nM	Human pulmonary endothelial barrier	Konya et al., 2013
	3–30 nM	Human platelet aggregation	Philipose et al., 2010
	800 nM/kg/day	Bone formation, mouse	Yoshida et al., 2002
ONO AE1-734	0.1 mg/kg/day	Colitis, mouse	Kabashima et al., 2002
PGE_1_-OH	10–1000 nM	Human dermal microvascular endothelial angiogenesis	Zhang and Daaka, 2011
TCS 2510	1 μM	Renal epithelial cell proliferation, mouse	Liu et al., 2012
	10 μM	GLP-1 release, mouse GLUTag cells	Coskun et al., 2013
γ-Lactam PGE analog 3	30–300 μg/kg	Bone fracture healing, rat	Kambe et al., 2012

**Table 2:** EP2/EP4 receptor agonists.

Name	Dose, concentration, affinity	Model, species	References
11-Deoxy-PGE_1_	100 nM–50 μM	Bone resorption, mouse	Sakuma et al., 2000
γ-Lactam PGE analog 2a	1.7 μg/kg	Bronchodilation, guinea pig	Xiao et al., 2008
γ-Lactam PGE analog 4	30–300 μg/kg	Bone fracture healing, rat	Kambe et al., 2012

**Table 3:** EP4 receptor-selective antagonists.

Name	Dose, concentration, affinity	Model, species	References
AH-23848	25–125 ng/g	Angiogenesis, mouse	Zhang and Daaka, 2011
BGC20-1531	pK(B) 7.6–7.8; 1–10 mg/kg i.v.	Cerebral and meningeal arteries, carotid blood flow, human	Maubach et al., 2009
CJ-023,423	56–97 mg/kg	Bone destruction and inflammation in arthritis, rat	Okumura et al., 2008
CJ-042,794	10–30 mg/kg	LPS-induced TNF-α production in whole blood, human	Hatazawa et al., 2007 and Murase et al., 2008
Diphenyloxazole 8	K_i_ = 0.3 nM	[^3^H]PGE_2_ binding studies, IgE synthesis in B cells, mouse	Hattori et al., 2005
ER-819762	10–100 mg/kg/day	Arthritis, mouse, rat	Chen et al., 2010
GW627368x	1 μM	Eosinophils, human	Luschnig-Schratl et al., 2011
	1 μM	Pulmonary vein, human	Foudi et al., 2008
	10 μM	Platelet aggregation, human	Philipose et al., 2010
L-161982	300 nM	Macrophages, human	Takayama et al., 2002
MF191	1 mg/kg	Bladder overactivity, rat	Chuang et al., 2012
MF498	1–30 mg/kg	Analgesia in rheumatoid arthritis, rat Joint pain in osteoarthritis, guinea pig	Clark et al., 2008
ONO AE2-227	300 ppm in diet	Reduced formation of preneoplastic lesions, mouse	Mutoh et al., 2002
ONO AE3-208	10 mg/kg/day	Colitis, mouse	Kabashima et al., 2002
	100–300 nM	Eosinophil, human	Konya et al., 2011 and Luschnig-Schratl et al., 2011
	10 nM	Aortic rings, mouse	Hristovska et al., 2007
	300 nM	Pulmonary endothelial barrier, human	Konya et al., 2013
	100–300 nM	Platelet aggregation, human	Philipose et al., 2010
